# Characterization of Mutations Associated with Streptomycin Resistance in Multidrug-Resistant *Mycobacterium tuberculosis* in Zambia

**DOI:** 10.3390/antibiotics10101169

**Published:** 2021-09-26

**Authors:** Precious Bwalya, Tomoyuki Yamaguchi, Eddie Samuneti Solo, Joseph Yamweka Chizimu, Grace Mbulo, Chie Nakajima, Yasuhiko Suzuki

**Affiliations:** 1 Division of Bioresources, International Institute for Zoonosis Control, Hokkaido University, Sapporo 001-0020, Japan; preciousbwalya@czc.hokudai.ac.jp (P.B.); tomoyuki15841@gmail.com (T.Y.); chizimu@czc.hokudai.ac.jp (J.Y.C.); 2 Department of Pathology and Microbiology, University Teaching Hospital, Ministry of Health, Lusaka 10101, Zambia; samuneti2@yahoo.com (E.S.S.); gmbulo@yahoo.co.uk (G.M.); 3 Zambia National Public Health Institute, Ministry of Health, Lusaka 10101, Zambia; 4 International Collaboration Unit, International Institute for Zoonosis Control, Hokkaido University, Sapporo 001-0020, Japan

**Keywords:** *Mycobacterium tuberculosis*, streptomycin, multidrug-resistant tuberculosis (MDR-TB), spoligotype, Zambia

## Abstract

Streptomycin (STR) is recommended for the management of multidrug-resistant tuberculosis (MDR-TB). Streptomycin resistance-conferring mutation types and frequency are shown to be influenced by genotypes of circulating strains in a population. This study aimed to characterize the mutations in MDR-TB isolates and examine their relationship with the genotypes in Zambia. A total of 138 MDR-TB isolates stored at the University Teaching Hospital Tuberculosis Reference Laboratory in Zambia were analyzed using spoligotyping and sequencing of STR resistance-associated genes. Streptomycin resistance was observed in 65.9% (91/138) of MDR-TB isolates. Mutations in *rpsL*, *rrs,* and *gidB* accounted for 33%, 12.1%, and 49.5%, respectively. Amino acid substitution K43R in *rpsL* was strongly associated with the CAS1_Kili genotype (*p* < 0.0001). The combination of three genes could predict 91.2% of STR resistance. Clustering of isolates based on resistance-conferring mutations and spoligotyping was observed. The clustering of isolates suggests that the increase in STR-resistant MDR-TB in Zambia is largely due to the spread of resistant strains from inadequate treatment. Therefore, rapid detection of STR resistance genetically is recommended before its use in MDR-TB treatment in Zambia.

## 1. Introduction

Tuberculosis (TB) caused by *Mycobacterium tuberculosis* is a global public health concern ranked among the top 10 causes of mortality. In 2019, an estimated 1.2 million deaths among HIV negative and 208,000 deaths among HIV-positive people were attributed to TB [1]. Zambia is among the 30 countries with the highest TB burden in the world. The emergence of drug resistance raises even more serious concern as it poses a threat to the strides made in controlling the disease. Efforts to reduce the incidences of drug resistance, particularly multidrug-resistant tuberculosis (MDR-TB), which is resistant to rifampicin (RIF) and isoniazid (INH), have led to a steady decline of global cases. On the contrary, in Zambia, the incidences of MDR-TB show an upward trend. From 2014 to 2019, MDR-TB has risen from 0.3% to 2.4% in new cases and 8.1% to 18% in previously treated cases [2]. This observed increase in MDR-TB in Zambia can be attributed to an increase in coverage of resistance testing and/or the emergence and spread of multidrug-resistant *M. tuberculosis* (MDR-*Mtb*) due to inadequate control measures.

Previous guidelines in Zambia recommended that drug susceptibility testing (DST) should be performed on all retreatment cases and suspected treatment failures. However, adherence to the guidelines was low [3], leading to the usage of drugs without confirmation of susceptibility testing. This practice could have led to the undetected emergence and silent spread of drug-resistant TB. In a previous study, it was demonstrated that the increase in MDR-TB in Zambia is not the result of transborder transmission but local emergence and possible spread within Zambia [4,5]. The mismanagement of MDR-TB could further the evolution of resistance to other anti-TB drugs. Recently, the first confirmed case of extensively drug-resistant TB (XDR-TB) was reported in Zambia [6], stressing the importance of selecting effective drugs for treatment to avoid amplification of resistance to other drugs and prevent transmission of MDR-*Mtb*. The adoption of tools that rapidly detect resistance could guide the selection of effective drugs and help control MDR-TB.

Streptomycin (STR) is one of two aminoglycosides recommended in TB treatment. It is currently recommended for limited use as a second-line drug in personalized treatment regimens of MDR-TB patients [7]. With the observed increased drug resistance in Zambia, STR still has a significant role in MDR-TB treatment. It is thus important to assess the level of STR resistance among MDR-TB patients for whom the drug is currently recommended and understand the molecular mechanism of resistance. This will help to assess the usefulness of STR in MDR-TB treatment and of a rapid molecular-based tool for the determination of STR resistance in Zambia.

Streptomycin induces the mistranslation of mRNA to protein by tightly binding to 16S rRNA and ribosomal protein S12 encoded by *rrs* and *rpsL,* respectively [8]. Mutations in *rpsL* are associated with high-level STR resistance, while mutations in the 530 loop and 912 region of *rrs* are associated with intermediate STR resistance [9]. In addition, mutations in *gidB* encoding a methyltransferase enzyme responsible for methylating a G nucleotide at position 518 of the *rrs* are known to confer low-level STR resistance [10,11]. These mutations in *gidB* are, at times, misclassified as susceptible at the recommended critical concentration of 1 µg/mL and may co-occur with high-level resistance-conferring mutations to amikacin, the only other aminoglycoside recommended in TB treatment [12]. Moreover, studies have revealed a difference in the types of mutations in different geographical locations and among different genotypes of *M. tuberculosis*. Therefore, it is important to investigate mutations conferring STR resistance in local settings, and this information could assist in the development and adoption of rapid diagnostic tools and, consequently, in the control of MDR-TB. This study is aimed at screening and characterizing mutations in *rpsL, rrs,* and *gidB* that confer resistance to STR and examine their association with the circulating genotypes in Zambia.

## 2. Results

### 2.1. Correlation between Genotypes and STR Resistance

The 138 MDR-*Mtb* analyzed included 69 (50%) isolates belonging to the LAM family, 34 (24.6%) to the CAS family, 19 (13.8%) to the T family, 6 (4.3%) to the H family, 7 (5.1%) to the X family, 2 (1.4%) to the EAI family, and 1 (0.7%) to the S family (Table 1). STR resistance was observed in 65.9% (91/138) of the MDR-TB isolates. Out of all the genotypes analyzed, CAS1_Kili (SIT 21) clade was more likely to have STR resistance compared to other spoligotype clades (OD: 3.9, *p* = 0.009). STR resistance was less likely to occur in LAM1 (SIT 20) (OD: 0.12, *p* = 0.0005).

### 2.2. Mutations in rpsL

Three types of amino acid substitutions (K43R, K88Q, and K88R) associated with mutations were identified in *rpsL* in 33% (30/91) of STR-resistant MDR-TB isolates (Table 2). K43R was observed in 70% of isolates with mutations in *rpsL,* followed by K88R at 26.7% and then K88Q at 3.3%. A silent mutation C117T (T39T) was observed in two isolates (Table S1, Supplementary Materials).

### 2.3. Mutations in rrs

Eleven STR-resistant isolates harbored four different types of mutations in *rrs,* including A514C, C517T, A906G, and A907C (Table 2). Mutations in *rpsL* and *rrs* were mutually exclusive and observed in resistant isolates only.

### 2.4. Mutations in gidB

Fifteen different nucleotide substitutions and five deletions were observed in *gidB* in 45 resistant and 12 susceptible isolates (Table 2). Ten mutations were exclusively found in resistant isolates. Three silent mutations at positions C12T (I4I), G211C (V110V), and A615G (A205A) were detected (Table S1). STR-resistant isolates with *gidB* amino acid substitution G71R and A183V were found to harbor additional mutations in *rrs* (A906G and C517T), respectively. A mutation involving two nucleotide substitutions (T611A; C612A) producing a stop codon at 204 was observed in five isolates. 

The nucleotide polymorphism T47G (L16R) was observed exclusively in the LAM genotype, while A615G (A205A) was observed in the CAS and EAI genotypes. Excluding the genotype-specific L16R, mutations with amino acid substitutions in *rpsL* and *gidB* were mutually exclusive.

The proportion of STR-resistant isolates with mutations in *gidB* was 49.5%. Eight STR-resistant isolates did not have mutations in any of the analyzed genes. 

### 2.5. Association of Genotype and Mutations in rpsL, rrs and gidB

A significant correlation was found between CAS1_Kili genotype and a mutation at codon 43 in *rpsL* (Odds ratio = 17.6, *p* value < 0.0001*)* (Table 3). Mutations in *gidB* were significantly correlated with the LAM genotype (*p* < 0.05). Clustering of isolates based on SIT number and STR resistance-conferring mutations was observed (Supplementary Material Table S1). 

### 2.6. Molecular Determination of STR Resistance 

The sensitivity of sequencing prediction of STR resistance using *rpsL* was 33%, while that of *rrs* was 12.1%, and *gidB* was 49.5% (Table 4). All three genes had an improved combined sensitivity of 91.2% and 74.5% specificity. 

## 3. Discussion

Streptomycin was used as a first-line drug in Zambia until the late 1990s [13,14]. In the early 2000s, it was recommended for the management of retreatment cases [15]. However, its prolonged use as a first-line drug in new and retreatment cases could have led to the emergence of STR resistance. This study is the first to describe mutations conferring STR resistance in MDR-*Mtb* and their relationship with the circulating genotypes in Zambia. 

A high proportion (65.9%) of STR resistance was observed among the MDR-*Mtb* in this study, which was lower than that observed in Uganda (83.9%) [16]. The high proportion of STR resistance in Uganda was estimated from a national surveillance program and thus reflects the national prevalence. In this study, however, isolates were sampled from a limited population. This signifies the importance of rapidly determining the STR susceptibility profile before its inclusion in the individualized MDR-TB regimen in Zambia [17]. 

The majority of isolates in this study belong to the LAM family (50%), with the LAM11_ZWE (SIT 59) clade being predominant, followed by LAM1 (SIT 20). Thus, LAM11_ZWE remains the major circulating *M. tuberculosis* genotype in Zambia [18,19]. The second most dominant genotype was CAS1_Kili (SIT 21) clade (24.6%). Previous studies had reported few isolates belonging to LAM1 (SIT 20) and CAS1_Kili (SIT 21) clades, suggesting a potential recent expansion of these strains. Compared to other clades, CAS1_Kili (SIT 21) had higher odds of STR resistance (Odds ratio 3.9, *p* value = 0.009) (Table 1). Conversely, LAM1 (SIT 20) clade was less likely to develop STR resistance (Odds ratio 0.12, *p* value = 0.0005). 

Mutations in *rpsL* were exclusively found in 33% of resistant isolates in our study. This was lower than the more than 65% frequency reported in regions with a high prevalence of the MDR-*Mtb* Beijing genotype (Lineage 2), such as Myanmar and China [20,21]. In contrast to these regions, Lineage 4 is predominant in Zambia, particularly the LAM genotype. Our results were consistent with reports from regions where the Beijing genotype circulation in the population is low, and *rpsL* mutations among MDR-*Mtb* isolates account for less than 50% of STR resistance [9,22,23].

Notably, in the present study, amino acid substitution K43R of *rpsL* was significantly associated with CAS1_Kili. Analysis of 7346 MDR, pre-XDR, and XDR isolates publicly available in the web-based database TB-Profiler revealed that Lineage 2 is more likely to acquire K43R amino acid substitution for STR resistance than any other genotype (Odds ratio 6.42, *p* value < 0.0001) (Table S3) [22]. However, this trend was not observed in Lineage 3, to which CAS1_Kili belongs. Consequently, the observed high proportion of the CAS strain harboring K43R in *rpsL* in this study indicates that, in addition to being a low fitness cost mutation [23], there is possibly clonal expansion of an *M. tuberculosis* strain with this mutation.

Mutations C517T and A906G in *rrs* have been reported in isolates with a minimum inhibitory concentration (MIC) between 10 µg/mL and 100 µg/mL, indicating that they sufficiently confer STR resistance [24]. In our study, an isolate with C517T mutation and two isolates with A906G mutation had additional mutations in *gidB.* One of the additional mutations occurred at codon 71, which is close to the GidB active site, and a mutation at this codon had been reported to have an MIC of 20 µg/mL [25,26]. Thus, the additional mutations may lead to increased levels of STR resistance. Mutations in *rrs* accounted for 12.1% of STR resistance, which is similar to China and South Korea but lower than Panama and Russia [27,28] (Table S2). 

Mutations in *gidB* encoding a methyltransferase result in non-methylation of G518 nucleotide of *rrs* and disruption of STR binding, leading to low-level resistance [29]. Though *gidB* is not a very long gene, its active site is made up of 16 codons, likely increasing the mutational target size [25] and leading to the observed polymorphic mutations. Among the mutations found in this study, 19 mutations had been previously described, while four were novel. The mutation C447G (S149R), previously detected in a resistant isolate with an MIC of 10 µg/mL [24], was found in one susceptible isolate in this study. Among the novel mutations found in this study included 25_88del (64 base pair deletion), C548T (A183V), 575_576delGC, and a double mutation T611A/C612A (204stop). Two of these (25_88del and 575_576delGC) were exclusively detected in STR-resistant isolates, confirming their role in conferring resistance. The double mutation T611A/C612A (204stop) was found in four resistant isolates and one susceptible isolate. The presence of resistance-conferring *gidB* mutations in some susceptible isolates indicates that the MGIT 960 drug susceptibility test method may misclassify low-level STR resistance. This can lead to the inclusion of STR in a regimen against an otherwise STR-resistant *M. tuberculosis* strain. These mutations, especially deletions and nonsense mutations, are expected to cause conformational changes in GidB, affecting its functionality [25]. Adoption of molecular testing would improve the detection and management of STR-resistant *M. tuberculosis*.

The non-synonymous mutation at codon 16 in *gidB* (L16R) has been associated with the LAM family in concordance with this study (*p* < 0.0001) [30]. The CAS and EAI families had the *gidB-*synonymous mutation A615G (A205A) observed in all isolates belonging to these families. This synonymous mutation has been reported in other lineages, suggesting that nucleotide A at position 615 in *gidB* in the H37Rv reference genome is a polymorphism specific to Lineage 4 [30,31]. 

A synonymous (V110V) mutation and a non-synonymous (G71R) mutation were detected exclusively in isolates belonging to the EAI genotype in this study. The V110V synonymous mutation has been described as an EAI genotype polymorphism [30]. In contrast, G71R may be a resistance-conferring genotype-independent mutation [31]. 

Clusters of two or more isolates were observed based on *rpsL*, *rrs*, *gidB*, *katG*, and *rpoB* mutations as well as spoligotype SIT (Table S1). Five clusters were observed in CAS1_Kili (SIT 21), with the largest cluster having 16 isolates and harboring K43R amino acid substitution in *rpsL*. It is postulated that CAS1_Kili (SIT 21) evolved in Tanzania and is dominant in that country, particularly in the city of Dar es Salaam [32,33]. This coastal city is of great economic importance as a trade harbor between Zambia and Indo-Pacific regions and could be the origin of CAS1_Kili found in Zambia. However, the CAS1_Kili (SIT 21) clade in Tanzania has not been associated with drug resistance [34]. It was possibly introduced to Zambia as an STR-susceptible clone before late-2000 when STR was being used as a first-line drug for TB treatment, and the acquisition of amino acid substitution K43R may have occurred in Zambia. We speculate that the progression to MDR emerged independently in some patients subsequent to infection with a K43R STR-resistant clone because these isolates had varied RIF resistance-conferring mutations in *rpoB*. Four additional clusters based on mutations in *gidB* and *rpoB* observed in CAS1_Kili (SIT 21) suggest several clones of STR-resistant MDR-*Mtb* CAS1_Kili (SIT 21) are spreading in Zambia (Table S1).

The spoligotype clade LAM1 (SIT 20), though not associated with STR resistance, could be considered one cluster of 16 isolates having the same spoligotype, *rpoB,* and *katG* mutations. Amino acid substitutions S450L in *rpoB* and S315T in *katG* were observed in 100% and 94.1%, respectively, in the LAM1 (SIT 20) isolates (Table S1). The frequency of *rpoB* S450L and *katG* S315T mutations in this clade exceeds the global estimates of 65% and 72%, respectively, among MDR-*Mtb* isolates [35,36]. These results support the clonal expansion of an STR-susceptible LAM1(SIT 20) clone. LAM1 is dominant in Angola and Namibia [37]. While there is limited information regarding *M. tuberculosis* drug resistance in Namibia, MDR-*Mtb* among retreatment cases was reported to be as high as 71% in Angola [38]. It is likely that an MDR-*Mtb* LAM1 clone susceptible to STR had been introduced in Zambia from Angola by displacement of people due to war. This could have been after STR was no longer used as a first-line drug, hence the low STR resistance in this cluster.

Among isolates belonging to the LAM11_ZWE (SIT 59) clade, clusters of five or more isolates harboring amino acid substitutions Y22H, V124G, and F204stop in *gidB* were observed (Table S1). The substitution V124G may have emerged first, followed by the acquisition of mutations conferring RIF resistance and are currently spreading in Zambia as STR-resistant MDR-*Mtb*, since two types of *rpoB* mutations were observed in this cluster. Isolates with Y22H and F204stop were restricted to Lusaka Province only. However, amino acid substitution V124G was found in isolates from Lusaka and Western Provinces, with the largest cluster (five isolates) being from the Western Province (Mongu District). This observation suggests that an *M. tuberculosis* strain with V124G may be disseminating to other areas from the Western Province.

The clustering of isolates in this study was not unique to a specific genotype but was observed in almost all genotypes, indicating that the increase in MDR-TB in Zambia is largely being driven by the spread of MDR-*Mtb* clones. This is further supported by the isolation from a nine-year-old patient, an isolate belonging to a cluster harboring a *gidB* G37R amino acid substitution, suggesting recent transmission (Table S1). The clustered clones had the same spoligotype SIT number as well as *rpoB*, *katG,* and *gidB* mutations. Though these mutations in *rpoB* and *katG* are frequently observed, mutations in highly polymorphic *gidB* permit the inference of clones even in the absence of spoligotype results. Outbreaks of MDR-*Mtb* characterized by mutations in *gidB* are not unprecedented [39]. Therefore, *gidB* sequencing may be useful in checking MDR-*Mtb* transmission. 

In this study, the sensitivity of mutations in *rpsL*, *rrs,* and *gidB* to predict STR resistance was 91.2%, which is similar to a report from China (94.6%) but higher than those reported in Myanmar (83.7%) and South Korea (84.3%) [20,21,39]. However, the sensitivity of *rpsL* in this study was lower (32.6%) when compared to these countries (Table S2). This could be attributed to the difference in the genotypes circulating in the different countries. Studies have shown that *gidB* mutations are significantly correlated with Lineage 4 while *rpsL* mutations are significantly correlated with Lineage 2 [12,40]. Accordingly, the observed higher sensitivity of *gidB* mutations (49.5%) compared to other genes in predicting STR resistance in this study could be attributed to the high number of isolates belonging to Lineage 4 (102/138). In contrast, in China and Myanmar, the majority of STR-resistant isolates belonged to Lineage 2 (the Beijing family), supporting the observed association between *rpsL* mutations and Lineage 2. This indicates that the genotype diversity of *M. tuberculosis* may influence the types of STR resistance-conferring mutations [41]. 

A limitation of this study was the non-inclusion of polyresistant and pan-susceptible isolates. Therefore, we could not confirm whether the observed mutations are also present in those groups of isolates in Zambia. 

## 4. Materials and Methods

### 4.1. Samples and DST

One hundred and thirty-eight isolates were randomly sampled from among multidrug-resistant *M. tuberculosis* isolates archived between 2011 and 2017 at the University Teaching Hospital Tuberculosis (UTH-TB) laboratory in Lusaka. The laboratory is a reference culture and DST facility covering the Eastern, Lusaka, and Western Provinces of Zambia. Demographic data and phenotypic drug susceptibility test results were obtained from the laboratory information system. 

Drug susceptibility testing was performed by the laboratory following the manufacturer’s manual (BD BACTEC^TM^ MGIT^TM^ 960 SIRE kit) (Becton, Dickinson and Company, Franklin Lakes, NJ, USA) for RIF, INH, ethambutol, and STR at concentrations 1.0 µg/mL, 0.1 µg/mL, 5.0 µg/mL, and 1.0 µg/mL, respectively. The BD BACTEC M960 machine was used for incubation.

### 4.2. DNA Extraction, PCR Amplification and Sequencing of rpsL, rrs, and gidB Genes

The extraction of DNA was done by heating at 95 °C for 15 min followed by overnight freezing. The two steps were performed twice. Next, an equal amount of TE buffer (10mM Tris-HCL pH 8.0, 1 mM EDTA pH 8.0) was added and stored at −20 °C until use.

PCR amplification and DNA sequencing were carried out using the primers previously described (Table 5) [20]. The purified, amplified DNA was sequenced with the Big Dye Terminator v3.1 (Thermo Fisher Scientific, Waltham, MA, USA) using a 3500 Genetic sequencer. Sequence results were aligned to H37Rv (NC_000962.3) using BioEdit sequence alignment editor software [42]. In addition, RIF and INH resistance-conferring mutations obtained in a previous study were used to confirm MDR [4].

### 4.3. Spoligotyping

Spoligotyping was conducted as previously described [5,43]. Briefly, the direct repeat region was amplified by PCR using primers DRa GGTTTTGGGTCTGACGAC and DRb CCGAGAGGGGACGGAAAC, and the resulting products were hybridized to 43 spacer-specific oligonucleotide probes on a membrane. The resulting spoligotype pattern was converted to binary and octal formats. The later format was compared with SpolDB4 to determine the spoligo-international type (SIT) and spoligotypes [44].

### 4.4. Statistical Analysis

Chi square (*X*^2^) and Fisher’s exact tests were used to determine the association between mutations and genotypes. Odds ratios and simple proportions were used to describe the data. Statistical significance was set at *p* value < 0.05. Statistical software IBM SPSS 26 was used for analysis.

## 5. Conclusions

This study has highlighted the high level of STR resistance among MDR-*Mtb* in Zambia. Mutations in *rpsL*, *rrs,* and *gidB* can adequately predict STR resistance in Zambia. Therefore, gene sequencing can be used to determine STR susceptibility before its inclusion in a personalized treatment regimen. This study has also shown that the sequencing of drug resistance-conferring mutations can be used to profile the drug resistance evolutionary history of isolates and detect transmission. The study also highlights that the rise in MDR-TB could be attributed to the possible spread of several clones of MDR-*Mtb* with resistance to STR in Zambia. To halt the spread, the development and adoption of a rapid molecular tool for STR resistance testing, prompt initiation of effective therapy, and the implementation of mask-wearing by all TB patients in high-risk environments are recommended.

## Figures and Tables

**Table 1:** Correlation between genotypes and phenotypic STR susceptibility.

Spoligotype		Streptomycin Resistant		Streptomycin Susceptible		Significance	
SIT	Clade	*n* = 91	Proportion (%)	*n* = 47	Proportion (%)	Odds Ratio (95%CI)	*p* Value
21	CAS1_Kili	29	31.9	5	10.6	3.9 (1.4 to 11.0)	0.009
59	LAM11_ZWE	21	23.1	8	17.0	1.5 (0.6–3.6)	0.4096
20	LAM1	4	4.4	13	27.7	0.1 (0.04–0.39)	0.0005
815	LAM11_ZWE	10	11.0	3	6.4	1.8 (0.5–6.9)	0.39
53	T1	8	8.8	4	8.5	1.0 (0.3–3.6)	0.96
Orphan	LAM11_ZWE	4	4.4	1	2.1		
137	X2	3	3.3	4	8.5		
52	T2	3	3.3	2	4.3		
Orphan	H1	2	2.2	2	4.3		
42	LAM9	1	1.1	2	4.3		
4	H3/T1	0	0.0	1	2.1		
Orphan	EAI	2	2.2	0	0.0		
34	S	1	1.1	0	0.0		
50	H3	1	1.1	0	0.0		
73	T	0	0.0	1	2.1		
317	T2	1	1.1	0	0.0		
811	LAM11_ZWE	1	1.1	0	0.0		
2173	LAM11_ZWE	0	0.0	1	2.1		

**Table 2:** Distribution of mutations in *rpsL*, *rrs,* and *gidB* among STR-resistant and susceptible isolates.

*rpsL* (DNA)	RpsL (Protein)	*rrs*	*gidB* (DNA)	GidB (Protein)	STR Resistant	STR Susceptible
A128G	K43R				21	0
A263G	K88R				8	0
A262C	K88Q				1	0
		A514C			3	0
		C517T			2	0
		C517T	C548T	A183V	1	0
		A906G	G211C	G71R	2	0
		A907C			3	0
			25_88del	9fs	1	0
			T64C	Y22H	3	2
			98delG	34fs	3	1
			G109A	G37R	3	1
			112delC	39fs	4	1
			C223T	P75S	1	0
			G227A	G76D	1	0
			T242C	I81T	1	0
			T298C	F100L	1	1
			347delG	117fs	5	2
			T371G	V124G	5	1
			C401A	A134E	1	0
			G412C	A138P	3	1
			C447G	S149R	0	1
			T455C	L152S	2	0
			G469C	G157R	2	0
			575_576delGC	193fs	2	0
			T611A, C612A	204 Stop	4	1
	No mutations				8	35

**Table 3:** Distribution of *rpsL*, *rrs,* and *gidB* mutations among different genotypes.

*rpsL* (DNA)	RpsL (Protein)	*rrs*	*gidB* (DNA)	GidB (Protein)	CAS	LAM	H	T	S	X	EAI
A128G	K43R				16	2	1		1	1	
A263G	K88R					4	1	3			
A262C	K88Q					1					
		A514C				3					
		C517T				2					
		C517T	C548T	A183V		1					
		A906G	G330T	G71R							2
		A907C						3			
			25_88del	9fs				1			
			T64C	Y22H		5					
			98delG	34fs	4						
			G109A	G37R				4			
			112delC	39fs	5						
			C223T	P75S		1					
			G227A	G76D		1					
			T242C	I81T		1					
			T298C	F100L		2					
			347delG	117fs	3					4	
			T371G	V124G		6					
			C401A	A134E			1				
			G412C	A138P		4					
			C447G	S149R	1						
			T455C	L152S		2					
			G469C	G157R		2					
			575_576delGC	193fs	2						
			T611A, C612A	204 Stop		5					
	No mutations				3	27	3	8		2	
	Total				34	69	6	19	1	7	2

**Table 4:** STR resistance prediction by mutations in *rpsL*, *rrs* and *gidB.*

Correlation of Drug Resistance Genotype and Phenotype								
	Susceptible		Resistant		Sensitivity (%)	Specificity (%)	PPV (%)	NPV (%)
	Mutation	No Mutation	Mutation	No Mutation				
*rpsL*	0	47	30	61	33.0	100	100	43.5
*rrs*	0	47	11	80	12.1	100	100	37.0
*gidB*	12	35	45	46	49.5	74.5	78.9	43.2
*rrs or rpsL*	0	47	41	50	45.1	100	100	48.5
*rpsL or rrs or gidB*	12	35	83	8	91.2	74.5	87.4	81.4

**Table 5:** Oligonucleotides used in this study.

Gene	Primer Set	
*rrs*	Foward	GATGACGGCCTTCGGGTTGT
	Reverse	AGGCCACAAGGGAACGCCTA
*rpsL*	Foward	GGCCGACAAACAGAACGT
	Reverse	GTTCACCAACTGGGTGAC
*gidB*	Foward	CGCCGAGTCGTTGTGCT
	Reverse	AGCCTGGCCCGACCTTA

## Data Availability

The data presented in this study are available within the article and in supplementary tables. Publicly available data were also analyzed. This can be found here https://tbdr.lshtm.ac.uk/ (accessed on 30 March 2021 ).

## Supplementary Materials

The following are available online at https://www.mdpi.com/article/10.3390/antibiotics10101169/s1, Table S1: Contains all the data, Table S2: Comparison of *rpsL*, *rrs,* and *gidB* mutations among MDR-MTB streptomycin-resistant isolates from various countries, Table S3: The occurrence of K43R in various lineages among 7346 global MDR, pre-XDR, and XDR-MTB isolates.
