# Supplementary material for: Characterization of Mutations Associated with Streptomycin Resistance in Multidrug-Resistant Mycobacterium tuberculosis in Zambia
Source: Antibiotics (Basel). 2021 Sep 26;10(10):1169. doi: 10.3390/antibiotics10101169 (PMC8532810; doi:10.3390/antibiotics10101169)
Supplement: Supplementary file 1 [file antibiotics-10-01169-s001.zip › antibiotics-1367532-Supplementary Table S2 and 3.pdf]

Characterization of Mutations Associated with Streptomycin Resistance in Multidrug-Resistant *Mycobacterium tuberculosis* in Zambia

Supplementary Materials

Table S2. Comparison of *rpsL*, *rrs* and *gidB* mutations among MDR-MTB streptomycin resistant isolates from various countries

Comparison of mutations among MDR-MTB streptomycin resistant isolates in various countries (Number of isolates)						
**Gene**	**Zambia (91)** Current study	**Myanmar (141)** [20]	**China (92)** [21]	**Russia (390)** [27]	**Panama (47)** [26]	**South Korea (51)** [39]
rpsL	33.0	69.5	78.3	51	8.5	64.7
K43R	23.1	64.4	58.7	40.8	8.5	47.1
K88R	8.8	4	19.6	10.2	0	17.6
K88Q	1.1	0	0	0	0	0
rrs	12.1	3.4	9.8	44.3	66	9.8
rpsL or rrs	45.1	73	88	94.3	74.5	74.5
gidB	49.5	9.9	9.8	1.8	19	9.8
rpsl or rrs and/or gidB	91.2	83.7	94.6	94.6	-93.6	84.3

Table S3. The occurrence of K43R in various lineages among 7346 global MDR,pre-XDR and XDR-MTB isolates [24]

**Lineages**	**# of isolates**	**# of isolates with K43R**	**Proportion**	**Odds Ration**	**95% CI**	***p* value**
L1	272	16	0.06	0.11	0.06-0.18	**<0.0001**
L2	3154	1827	0.58	6.42	5.78-7.15	**<0.0001**
L3	559	117	0.21	0.47	0.38-0.57	**<0.0001**
L4	3308	595	0.18	0.23	0.21-0.25	**<0.0001**
L5	44	9	0.21	0.48	0.21-0.95	0.048
L6	5	1	0.2	0.46		
L9	1	0	0	4.80E-05		
M.bovis	3	2	0.67	3.73		
